# Combined cell and nanoparticle models for TOPAS to study radiation dose enhancement in cell organelles

**DOI:** 10.1038/s41598-021-85964-2

**Published:** 2021-03-24

**Authors:** Marc Benjamin Hahn, Julián Mateo Zutta Villate

**Affiliations:** 1 Bundesanstalt für Materialforschung und -prüfung, 12205 Berlin, Germany; 2 Institut für Experimentalphysik, Freie Universität Berlin, 14195 Berlin, Germany; 3 Universidad Nacional de Colombia, Medellín, Colombia; 4 Departamento de Física, Facultad de Ciencias, Pontificia Universidad Javeriana, Bogotá, Colombia

**Keywords:** Nanotechnology in cancer, Computational biophysics

## Abstract

Dose enhancement by gold nanoparticles (AuNP) increases the biological effectiveness of radiation damage in biomolecules and tissue. To apply them effectively during cancer therapy their influence on the locally delivered dose has to be determined. Hereby, the AuNP locations strongly influence the energy deposit in the nucleus, mitochondria, membrane and the cytosol of the targeted cells. To estimate these effects, particle scattering simulations are applied. In general, different approaches for modeling the AuNP and their distribution within the cell are possible. In this work, two newly developed continuous and discrete-geometric models for simulations of AuNP in cells are presented. These models are applicable to simulations of internal emitters and external radiation sources. Most of the current studies on AuNP focus on external beam therapy. In contrast, we apply the presented models in Monte-Carlo particle scattering simulations to characterize the energy deposit in cell organelles by radioactive ^198^AuNP. They emit beta and gamma rays and are therefore considered for applications with solid tumors. Differences in local dose enhancement between randomly distributed and nucleus targeted nanoparticles are compared. Hereby nucleus targeted nanoparticels showed a strong local dose enhancement in the radio sensitive nucleus. These results are the foundation for future experimental work which aims to obtain a mechanistic understanding of cell death induced by radioactive ^198^Au.

## Introduction

The application of metallic nanoparticles (NP) in cancer treatment is considered for different forms of radiation therapy^1,2^. Most prominently, they are evaluated for external beam therapy with ionizing radiation^3–5^. Additional therapeutic approaches include brachytherapy in combination with internally placed radioactive emitters^6^, photothermal therapy^7–10^, and their usage in the form of radioactive gold nanoparticles (AuNP) them self^11–16^. All these different forms of application have in common that they benefit from an increase of the local energy deposit in the surrounding of the NP upon interaction with ionizing radiation. This increase in energy deposit is caused by the higher scattering cross section of the metallic NP when compared to water or organic matter. The resulting increase of damaged biomolecules (*e.g.* DNA in the nucleus or mitochondria, proteins) is based on a locally increased production of reactive secondary damaging species such as Auger electrons, low energy electrons (LEE) and reactive oxygen species (ROS)^17–22^. To assess the effectiveness of AuNP in radiation therapy, their lethality towards different cell lines have to be evaluated. Especially their energy deposit characteristics in cell organelles, such as mitochondria, cell membrane and the nucleus, have to be predicted for the respective cell lines, to obtain a mechanistic understanding of experimentally obtained exposure-survival curves.

For effective therapies, it is of importance to increase the dose delivered to the tumor while sparing healthy tissue^23^. The AuNP discussed here, are considered for applications with solid tumors. The optimal delivery strategy depends on the tumor type which has to be treated^2^. Active targeting is a promising approach to specifically targets cancer cells through direct interaction between ligands and receptors of the cells. To distinguish them from healthy cells, ligands on the surface of NPs can be selected to specifically target overexpressed molecules which are only located on the surface of cancer cells^24,25^. The interaction between ligands and receptors can induce endocytosis, which allows internalization of AuNPs. Afterwards the AuNP can release their energy into tumor cells effectively, which leads to an enhancement of the locally absorbed dose^15^. The local dose enhancement effects of AuNP takes place until most of the secondary particles, originating from the radiation-NP interaction, thermalize^3,15^. Therefore, it is beneficial to not only introduce the NP into the cancerous tissue, but also to have them accumulate in the vicinity of the most radiation sensitive parts of the cell, *e.g.* nucleus or mitochondria^1,4^. However, plain AuNP tend to accumulate in the cytoplasm, while the preferential target for radiation is the nuclear DNA^4,26^. Thus, a promising way to enhance the effectiveness of radiation induced cell killing is to specifically target tumor cells and their nucleus^13,27^. This can be achieved by tailor made AuNP coatings from peptides, as recently demonstrated by Özçelik and Pratx^27^. They observed a 3.8-fold reduced cell proliferation for nucleus targeted AuNP during *in vitro* irradiations.

To study these effects, Monte-Carlo based particle-scattering simulations are an effective tool to predict energy deposit in irradiated cells. However, the presence of AuNP in irradiated cells can be modeled in different ways. For example, a *continuous model* assumes a homogeneous distribution of gold within the cytosol while neglecting the nanoscopic structure of the AuNP (*e.g.* geometry, radius). A *discrete-geometric model* where individual AuNP are placed within the cell, provides a more detailed picture together with information on small scale effects by variation of AuNP location, radii *etc.*^15^. Especially nanoparticles targeting the nucleus can be simulated properly only by a *discrete-geometric model*^1,27^. However, such a detailed description increases the computational cost due to the creation of more complex geometries, higher amount of simulated boundaries and less homogeneity. Here, we evaluate the (dis-)advantages of these different approaches to simulate the effects of AuNP on the dose distribution within a model system, and to provide guidance for future combinations of simulational and experimental work. Therefore, we extend the recently developed cell models within *Topas-nBio*^28^ by adding the possibility to explicitly include nanoparticles in the cell during Monte-Carlo based particle scattering simulations with *Geant4*^29–31^ and *Topas*^32^. Hereby, the nanoparticles can be randomly distributed in the cytosol or attached to the nucleus or mitochondria, emulating different treatment conditions. The newly presented extension will be applied to a model cell line, Chinese hamster ovary cells (CHO), and radioactive ^198^AuNP. The inclusion of ^198^AuNP in a generalized cell model was chosen since it provides, a computational demanding application for the the presented cell-nanoparticle extension, as well as a useful guidance for future experimental application of ^198^AuNP in different cell lines. The energy deposit characteristics of isolated radioactive ^198^AuNP in dependence on their intrinsic and extrinsic properties *e.g.* AuNP diameter, AuNP density, relative gold mass percentage and their clustering behavior were investigated in a previous study^15^. The results showed an increase of the energy deposit within 150 nm around the AuNP in dependence of the AuNP radius (2.5–20 nm). For the investigated AuNP clusters, the enhancement of the energy deposit increases locally with the relative gold mass percentage. However, to obtain information on the effects on a cell, their locations have to be considered. Therefore, we extend this previous work by a flexible cellular model to study the effects of randomly distributed as well as nucleus targeted AuNP. Hereby, we focus on the energy deposit to different cell organelles in dependence of AuNP location and the computational speed, which is of importance for the simulation of complex geometries and the possibility to evaluate a huge parameter space.

## Results

### The cell models

**Figure 1:**
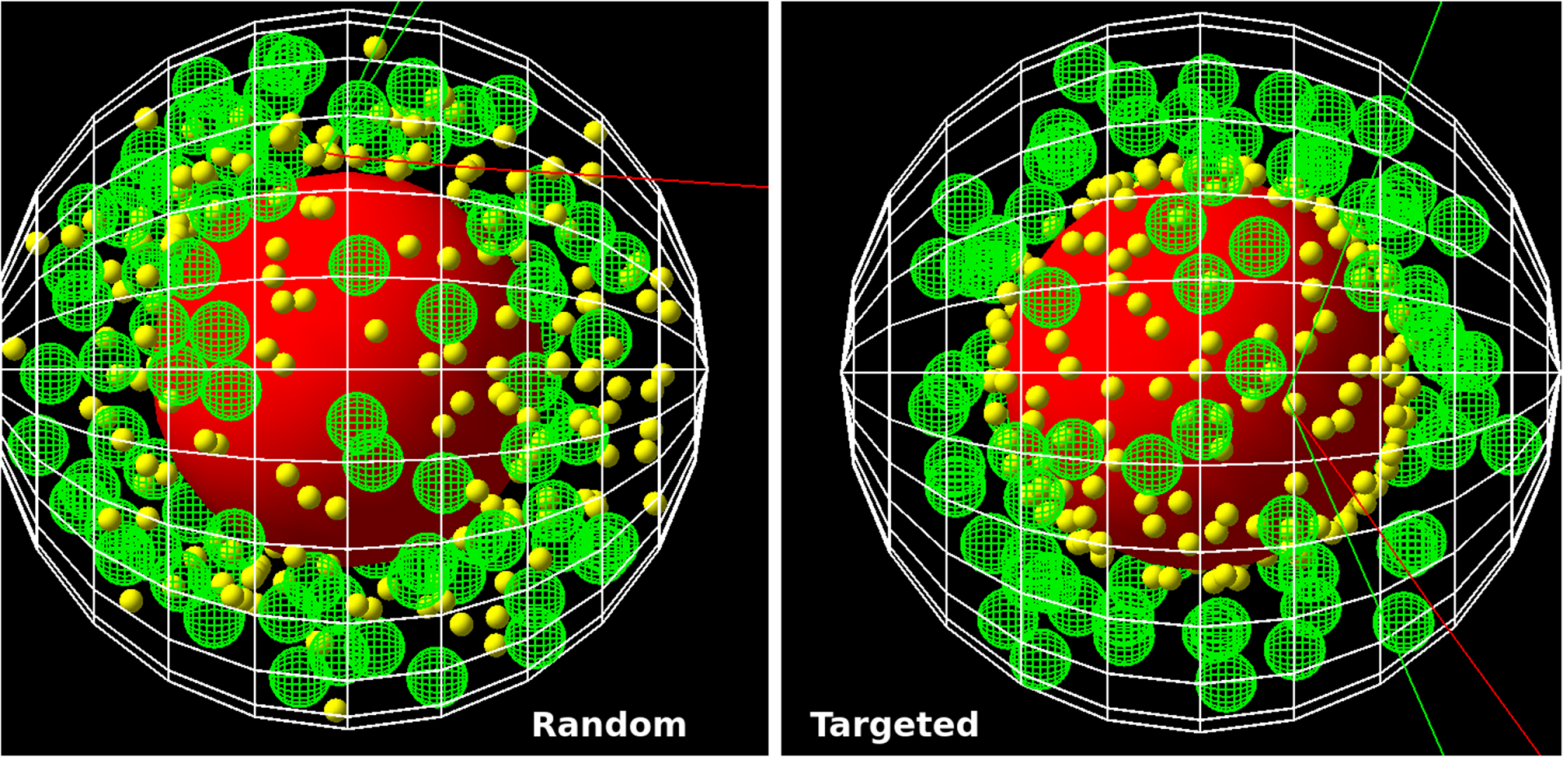
Visualization of the spherical cell model with the nucleus (red) and 90 mitochondria (green) generated by Topas (3.5)^32^ and the *TsSphericalCellSphericalNP* model with the dimensions as given in Table 2. For illustrative purposes two hundred AuNP (yellow) were generated randomly in the cytosol (left) or targeted to the nucleus surface (right) and their radius was set to 200 nm. Electron trajectories are shown in red, photons in green.

Combined cell and nanoparticle models were developed and are presented in this work. The cell models were implemented for *Topas*^32^ and *Topas-nBio* which provide an easy to use interface to the Monte-Carlo particle-scattering framework *Geant4*^29–31,33^. They share structures, ideas and naming conventions with the cell classes provided by *Topas-nBio*^28^. Both cell models can be obtained from our webpage^34^ and the continuously updated version from our *github* account^35^. The presented models have the capability to simulate radiation interaction with cells, including the nucleus, mitochondria, the outer cell membrane, as well as randomly placed nanoparticles within the cytosol (Fig. 1 left) and nanoparticles located at the surface of the nucleus (right) or mitochondria. 
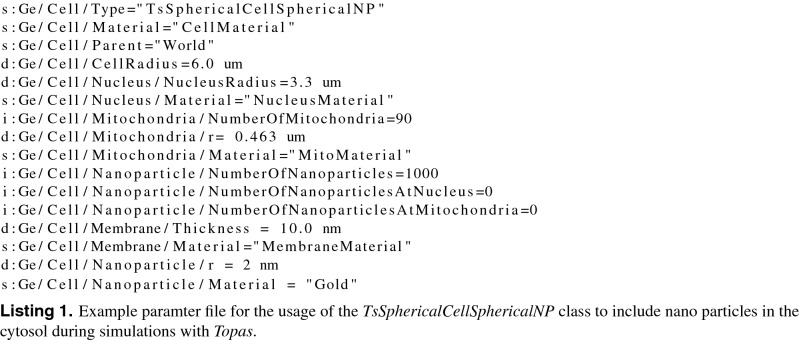

The determination of the dose and energy deposit in all these subvolume can be obtained from the simulations by the scorers provided. The models are easily applied by defining them as standard object within a *TOPAS* input parameter file. An example, showing the usage of this class is given in listing 1. A visualization of the *TsSphericalCellSphericalNP* model is shown in Fig. 1. From a computational point of view, two different realizations of the cell models were implemented. The first model enables the simulation of cell organelles in form of ellipsoids (*TsSphericalCellNP*). Here, the drawback is the need for computational expensive overlap checking. To enable faster simulations, a second version restricted to spherical NP and organelles was developed. The second version (*TsSphericalCellSphericalNP*) provides much faster overlap checking for high number of NP and organelles by analytical methods.

#### The TsSphericalCellSphericalNP model

The first model was optimized for the fast generation of spherical cell geometries (*TsSphericalCellSphericalNP*) including nanoparticles. It shares some structures, ideas and naming conventions with the cell classes provided by *Topas-nBio*^28^. Additionally, nanoparticles and an outer cell membrane can be simulated explicitly. Here, a new method for the placement and overlap checking of cell organelles and nanoparticles was implemented by the authors. It improves simulation speed and offers the inclusion of nanoparticles which reside at the surface of the cell nucleus or mitochondria. In this model only spherical nanoparticles and spherical cell organelles can be included. The restriction to spherical geometries enables a much quicker (analytical) check for overlaps of the randomly placed objects within the cell compared to the general approach valid for arbitrary geometries as implemented within *Topas* itself. The drawback is indeed the loss of the ability to use nanoparticles or organelles with other than spherical shapes.

#### The TsSphericalCellNP model

The second implementation provides a more general model which enables inclusion of ellipsoids within different cell geometries. This model (*TsSphericalCellNP*) was extended by the authors and is based on the *TsSphericalCell* class as provided by *Topas-nBio*^28^. Since the overlap checking is much faster with the *TsSphericalCellSphericalNP* class, when many objects are included in the cell, all simulations presented in the following were performed with this class.

### Comparision of the continuous and discrete-geometric AuNP models

The energy deposit in dependence of the Au mass percentage in the cell for simulations of the continuous model and randomly distributed discrete-geometric AuNP are summarized in Table 1. The absorbed dose after $10^5$ decays of a cell is about 32 Gy-35 Gy, which leads to an expected survival rate of less than 1%^36^.

**Table 1:** Energy deposit per decay (dec.) in the cell organelles for continuous (Cont.) and discrete-geometric (Disc.) models.

Model (unit)	r_AuNP_ (nm)	m_AuNP_ (%)	Cell (eV/dec.)	Cytosol (eV/dec.)	Nucleus (eV/dec.)	Mito. (eV/dec.)	Membrane (eV/dec.)	AuNP (eV/dec.)
Cont.	–	0.001	1810±2	1485±1	232±1	87.3±0.2	5.44±0.02	–
Cont.	–	0.01	1812±2	1488±1	232±1	87.1±0.2	5.42±0.02	–
Cont.	–	0.1	1814±2	1489±1	233±1	87.3±0.2	5.40±0.02	–
Cont.	–	1	1822±2	1497±2	232±1	87.5±0.2	5.44±0.01	–
Disc.	3	0.0024	1906± 8	1537±8	272±4	91±2	4.85±0.05	57±1
Disc.	4	0.0057	1913±15	1544±11	273±6	92±2	4.84±0.01	66±2
Disc.	5	0.011	1911±7	1543±6	273±6	92±2	4.81±0.05	74±1

Energy deposit in the cell and cytosol include the energy deposit in the AuNP. Energy deposit values for mitochondria (Mito.) and AuNP are given as sum over all objects.

**Figure 2:**
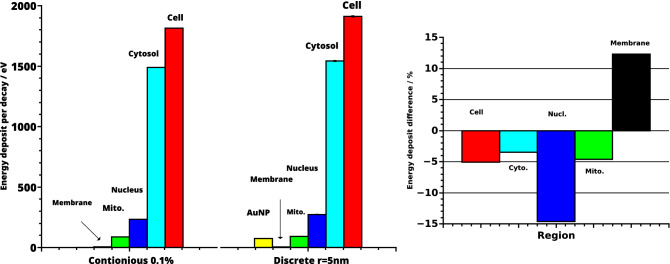
Left: Energy deposit per decay in the respective region of the cell for the continuous model with m_Au_ = 0.1% and the discrete-geometric model for r_AuNP_ = 5 nm. Energy deposit in the cell and cytosol include the energy deposit in the AuNP. The errorbars represent the standard deviation for n = 10 simulations each. They are in the size of the line thickness and therefore not visible. Right: Relative change of the energy deposit in the continuous model with respect to the discrete-geometric model.

For the continuous model the results show an increase of energy with increasing Au mass percentage and density in the cytosol from 0.001 to 1% of less then 2% deposit within the whole cell and it its parts. The uncertainties given are calculated from the standard deviation for n = 10 simulation runs, each with a different random number generator seed. In the discrete-geometric model variation of the results is increased, therefore no significant differences between the AuNP radii of 3 nm, 4 nm and 5 nm result were observed within the simulational uncertainties. In general, the total energy deposit in all parts, except of the membrane, is 4–15% lower when simulations are performed for the continuous compared to the discrete-geometric model (Fig. 2 right).

### Nucleus targeted nanoparticles

**Figure 3:**
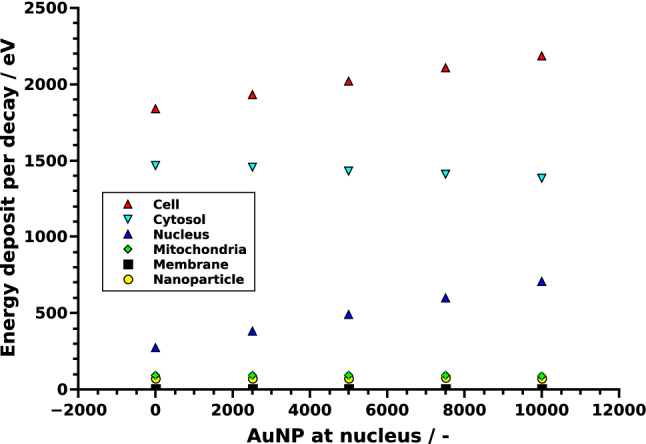
Energy deposit per decay in dependence of the AuNP location. For the whole CHO cell (red) and the cytosol (light blue), here excluding the AuNP (yellow), the nucleus (dark blue) and the 90 mitochondria (green) and the cell membrane (black). The errorbars are in the size of the symbols and therefore not visible. For details see the text.

**Figure 4:**
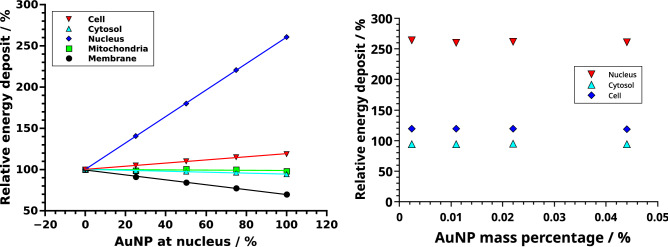
Left: Relative energy deposit ($E_{rel}(x)=100\,\%\, \cdot \, E(x)/E(0\,\%)$) in dependence of AuNP (r = 5 nm) located at the nucleus with respect to the case of all AuNP distributed randomly in the cytosol. The total amount of AuNP in the cell is constant (n = 10,000). The curves represent linear fits. Right: Relative energy deposit in the whole cell, cytosol and nucleus when all AuNP are located at the nucleus compared to randomly distributed AuNP for different mass percentages and radii. For details see the text.

The relative accumulation of AuNP at the nucleus was varied between 0 and 100%. The energy deposit in the whole cell as well as in the nucleus increased linearly (both $R^2>0.99$) with AuNP accumulation at the nucleus, while the deposit in the outer cell membrane decreased somewhat (Figs. 3,  4 left). The relative energy deposit ($E_{rel}(x)$) is the energy deposit caused by *x*% targeted AuNP with respect to Energy deposit without nucleus targeting AuNP (x = 0%). It is calculated by $E_{rel}(x)=100\,\%\, \cdot \, E(x)/E(0\,\%)$. Hereby *E*(*x*) is the energy deposit in the respective volumes of organelles caused by *x*% of the AuNP being present at the nucleus. $E(0\,\%)$ represents the case, where 100% of the AuNP were distributed randomly within the cytosol and 0% at the nucleus. When AuNP are exclusively located on the surface of the nucleus, the energy deposit within the nucleus increases to 260%. For this case the overall energy deposit in the cell increases about 20%. Effects on the mitochondria are negligible (Fig. 4). These location dependent dose enhancements are independent of the relative mass percentage of the AuNP as can be seen in Fig. 4 right. There, AuNP mass percentages between 0.0024% (10$^4$ AuNP with r = 3 nm—first datapoint) up to 0.044% (4$\times$10$^4$ AuNP with r = 5 nm—last datapoint) were simulated. Based on the results of our previous study^15^, the resulting enhancement efficiency, as shown in Fig. 4, can be expected to be similar for varying AuNP diameter when the same mass percentage is considered.

### Computational cost of the different modeling approaches

In general, the continuous model is much faster (4  threads, t$\approx$1 hour per run) than the discrete-geometric models (16  threads, t$\approx$1-5 days per run). Exact values depend on the interplay of many parameters such as the amount and size of NPs, number of organelles, and the total cellular volume. These differences in simulation speed are mostly due to the reduced complexity in the continuous model, in terms of geometries, boundaries between different materials and time needed to find initial decay positions. The generation of the geometries and the check for overlaps of subvolumes is much faster when performed analytically as implemented in the *TsSphericalCellSphericalNP* class compared to the *TsSphericalCellNP*. In both discrete-geometric models the time for the initial generation of the geometry, and especially the random placement of the NP in the cell, increases exponentially with the number of subvolumes included. Since the process of overlap checking runs on a single thread only it can take up a significant amount of the whole simulation time for high numbers of NP (> 100,000). Furthermore, the implementation of volumetric radioactive sources in *Topas* searches for random points within the the active volume (here, the cell) which contain the active material (the AuNP). Since the total volume of the cell is much bigger than the total volume of all AuNP, the search for decay position can take a significant amount of time when there are low numbers of particles in the cell. However, this applies only for radioactive sources and does not concern applications of the model to external beam therapy. We note here, that detailed statements about exact simulation speeds and their comparison depend on a multitude of factors. Thus, all these values should be interpreted as been rough approximations.

## Discussion

Metallic NP are introduced into cancerous tissue during radiation therapy to enhance the local dose. Hereby, it is beneficial when the NP accumulate in the vicinity of the radiation sensitive parts of the cell, *e.g.* the nucleus or mitochondria^1,4^. To study accumulation effects and analyze related experiments, *in silico* studies can provide detailed information about NP localization effects, which are otherwise inaccessible by experiments alone. Therefore, a detailed model of the sub-cellular geometries and NP is necessary, which was introduced above. The newly presented combined cell-nanoparticle model was implemented as a *TOPAS*-extension, which enables the study of radiation-matter interaction covering all types of radiation and energies applied in cancer therapy^32^. TOPAS was chosen since it is freely available upon request, and provides an easy to use interface to the complex *Geant4*-framework^29,33^. The extension was used in an exemplary application to the computationally demanding task simulating radioactive ^198^AuNP. We note that the application of the extension to external beam-therapy is straight forward and accessible. Here, simulation times are in the range from hours to days for a single run with 10$^5$ external photons.

Different simulations to characterize the energy deposit in cell organelles by radioactive ^198^AuNP were performed. Firstly, discrete-geometric AuNP were randomly placed in the cytosol of CHO cells (Fig. 1 left), representing the case where the AuNP do not target the nucleus. The comparison of these results with a simplified continuous model revealed deviations of up to 15% in the energy deposit values in the radiation sensitive nucleus (Fig. 2). This outcome can be rationalized when considering the local dose enhancement, which is only present in the discrete-geometric model. It is strongest within a range up to some ten nanometers around the AuNP. For example, within the first 25 nm around the source, the energy deposit density per decay is approximately twice as high for an AuNP with r = 5 nm, when compared to a point source. This enhancement of the local dose decreases quickly and vanishes at about 175 nm, as calculated in a previous study^15^. Thus, the AuNP have to reside as close as possible (preferentially nearer than approximately 100 nm), to the biologically relevant target (*e.g.* the cell nucleus or mitochondria)^37^. Further away, at distances of about 1 $\mu$m the energy deposit is only slightly (< 2%) increased by their presence. Furthermore, the average energy deposit in the cell increased linear with AuNP presence at the surface of the nucleus (Fig. 3). This can be understood by the longer electron and photon tracks throughout the cell. The energy deposit in the outer cell membrane decreases due to their on average higher distance to the nanoparticles and decay positions. When AuNP are exclusively located on the surface of the nucleus the energy deposit can reach up to 260% compared to the case of randomly distributed AuNP. Additionally, the overall dose in the cell increases about 20% (Fig. 4). When only 20% of the AuNP are located at the nucleus an energy deposit enhancement can still be observed. This is owed to the strong energy deposit enhancement around the AuNP, where the decay takes place. For example, from 50 nm to 150 nm distance to the AuNP surface, the energy deposit density decreases over tenfold^15^. Thus, the most important parameters for increasing the local energy deposit in the nucleus is the location of the AuNP within the cell. In case of radioactive AuNP, their specific activity, which is determined by the activation protocol used, has to be taken into account as well. It has to be noted, that the location dependent enhancement of the energy deposit per decay, does not depend on the Au mass percentage, as it was simulated for AuNP mass percentages up to 0.044% (Fig. 4 right). This shows, that at these AuNP concentrations most of the inelastic scattering events in Au happen within the AuNP where the initial decay event took place. The combination of these parameters offers the possibility to adjust and optimize the AuNP behavior for cellular uptake or metabolic behavior^15,38^. Especially selective permeability into the cell depending on AuNP size, their structure and functionalisation are of interest to specifically target tumors^39^. Furthermore, to predicts cell death or mutation, the rate of DNA double-strand breaks (DSB) has to be estimated in dependence of the local energy deposit in the nucleus. Recent theoretical studies estimated a threshold value for DSB induction as $E_{t}^{DSB}=(76-103.54)\,eV$^40^. A combined experimental and simulational study showed that an energy deposit of about $E_{1/2}^{DSB}=72\,eV$ in the target volume of the respective sugar-phosphate backbone of DNA leads with a 50% probability to a DSB^41^. This work accounted for direct radiation effects by ionization of DNA and the indirect effects mediated by ROS produced from water radiolysis. Based on this data a microscopic target-model can be implemented to estimate DSB induction, and related biological response of the cell afterwards. The flexibility of the cell model presented here, allows for an easy extension in future work, and provides a powerful tool to benchmark theoretical models against experimental datasets from different cell lines. We note here, that the “biological response” of the cells, in terms of radiation sensitivity and repair efficiency depends on a multitude of factors, which have to be considered for each cell line separately. Another important point is the possible toxicity of AuNP. Gold nanoparticles have been widely used in current medical and biological research, much experimental work has been done which confirms the non-toxicity of AuNPs, and are considered comparatively safe^42^. Especially when compared to other treatment agents, as used in chemotherapy. On the other hand, they are not completely bio-inert and bio-compatible; since toxicity is directly related to various factors such as size, shape, and surface chemistry^42^. Toxicity has been observed at high concentrations greater than 10 $\mu$g/mL^42^. However, concerning AuNP with diameters comparable to the present study, Alkilany *et al.*^43^ demonstrated that for d = 1.4 nm (up to 0.4 $\mu$M, 72h) to 18 nm (0.001-0.25 $\mu$M, 72h) spherical gold nanoparticles were non-toxic from *in-vitro* study. Besides, spherical gold nanoparticles with 1.9 nm diameter were found to be non toxic, when administered to mice for a tumor therapy^23^. Furthermore, radioactive gold colloids have already been effectively used in local radioisotope cancer therapy in humans^44^. A detailed discussion of all these factors is beyond the focus of this study.

In future studies, where the energy deposit with nanometer accuracy is of interest, special care has to be taken to choose appropriate scattering models^45^. When track-structure code, where every scattering event is explicitly simulated, is available for the materials under investigation, its usage is recommended. As an alternative, when only condensed history codes are available, it has to be taken care that simulation parameters, such as production and range cuts, are sufficiently low to minimize the associated uncertainties^46^. For example, the path length of secondary electrons with energies below 100 eV can vary up to 10 nm in dependence of the medium, scattering cross sections and scattering models applied^47^. This has to be considered when more detailed statements about energy deposit distribution within chromosomes or even smaller structures are made^46^. However, in this study, we focus on the nucleus which has a diameter of above 6 $\mu$m (Table 2), thus, the resulting differences are negligible for the analysis preformed here. For the AuNP, which are indeed much smaller, simulations should preferentially be performed with track-structure code. Therefore the simulations of AuNP will benefit greatly from the new scattering models for gold implemented by Sakata *et al.* when they become available in Geant4 and Topas^48–50^.

## Summary and outlook

In this work we have presented combine cell-nanoparticle models for *Topas/Geant4* to simulate the local dose enhancement effects caused by the presence of nanoparticles in the cytoplasm, as well as on the nucleus surface of cells. The models were applied to determine the energy deposit caused by the presence of radioactive AuNP nanoparticles in CHO cells. Simulations were performed for continuous and a discrete-geometric AuNP models. These discrete-geometric nanoparticle models enable the simulation of non-homogeneous distribution of AuNP within the cell. The energy deposit in the cytosol, mitochondria and nucleus were determined in dependence on AuNP locations. Future work will extend the presented cell models to predict DNA strand-break induction within the cell. This extension will be based on a recently developed DNA damage mode, which accounts for direct radiation effects by ionization of DNA and the indirect effects mediated by reactive-oxygen species produced from water radiolysis. Another possible extension towards more realistic cellular models, is the inclusion of clustering behavior of mitochondria within the cytosol. In conclusion, it was shown that the type of simulation model and AuNP location within the the cell strongly influences the energy deposit in different organelles. Thus, AuNP which target the nucleus or mitochondria of cancerous tissue have the potential to greatly enhance damage in tumours while decreasing side effects on healthy tissue.

## Methods

### Particle scattering simulations

To obtain the energy deposit in different cell organelles Monte Carlo simulations (MCS) based on the *Geant4* MCS framework (10.06)^29–31^ in combination with the *Topas* (3.5)^32^ interface and the *Topas-nBio*^28^ extensions were performed. The radioactive decay of ^198^Au, the production of secondary particles, and their interaction with the surrounding matter was simulated. In each simulation $10^5$ decays of the AuNP (19.32 g/cm^3^) were simulated by the *g4radioactivedecay* and *g4decay* modules. All other scattering processes were simulated with the processes provide by the *g4emstandard_opt4* physics lists and a range cut for all particles of 2 nm was applied. This physics list provides the most accurate standard and low-energy models for electron scattering, with a decreased range factor (0.08) to improve the accuracy and the atomic de-excitation modules were set to ignore the cuts^51^. The standard models for atomic de-excitation, Auger electron emission, Auger cascade and fluorescence were enabled. During the simulations the position of the radioactive decay was chosen randomly within the active material. It has to be taken into account, that the scattering-models applied in the AuNP region have a recommended low energy limit of 100 eV. This corresponds to a range of secondary electrons of about 100 nm^52,53^, which puts some constrains on the accuracy of simulations of nanometer sized structures. When possible, simulations should preferentially be performed with track-structure code which enables step-by-step simulations of the scattering interactions, and results in a higher accuracy compared to condensed-history code^50,54^. Thus, this situation will improve in the future when the newly implemented scattering models for gold as implemented by Sakata *et al.* become available in future releases of Geant4 and Topas^48–50^. As a model system for testing, CHO cells were selected, since they are readily available and well established organism used in pharmaceutical production and radiation research^36,55,56^. Here we note that the amount of mitochondria within the CHO cells reported varies between approximately 60–200, in dependence on the counting methods applied^57,58^. Their geometrical parameters and chemical composition used throughout the simulations are summarized in Table 2.

**Table 2:** Geometrical parameters of CHO cells.

Type	r	H	C	N	O	S	P	Au
Unit	nm	%	%	%	%	%	%	%
Cytosol	6000	10.25	12.25	4.25	73.25-72.25	0.00	0.00	0-1
Nucleus	3300	10.60	9.00	3.20	74.20	0.40	2.60	0
Mitochondria	463	10.60	9.00	3.20	74.20	0.40	2.60	0
Membrane	10	10.25	12.25	4.25	73.25	0.00	0.00	0

The dimensions of cytosol, nucleus and mitochondria are given as radius. The membrane thickness is listed as diameter^59^. The chemical composition of the different parts of the cell is given in mass percent and density of 1.0 g/cm^3^^60^ Ninety mitochondria were simulated^57^.

### The discrete-geometric AuNP models

For the discrete-geometric models (We note here, that the term discrete refers to the explicit simulation of the AuNP within the medium, and is not to be confused with “discrete transport” in particle-scattering Monte-Carlo simulations) the simulations were performed with 10^4^ NP per cell with radii of 3 nm, 4 nm and 5 nm. These values correspond to gold mass percentages in the cell of $2.4\cdot 10^{-3}\,\%$, $5.7\cdot 10^{-3}\,\%$ and $1.1\cdot 10^{-2}\,\%$, respectively. The AuNP located in the cytosol are randomly distributed. The AuNP attached to the nucleus or the mitochondria are placed in direct contact with the respective surface, whereby the angles describing the location on the surface are chosen randomly. When simulations of mitochondria targeted NP are performed, which is not the case in the examples presented above, the respective mitochondria are chosen for each AuNP independently and randomly with equal probability. To assess the effect of nucleus targeted AuNP, we simulated a constant amount AuNP of whom a certain amount was distributed randomly in the cytosol or at the surface of the nucleus. Hereby the amount at both locations were varied between 0 and 100%.

### The continuous Au model

For comparison, the simulations for continuous distribution of Au within the cytosol were performed with the *TsSphericalCellSphericalNP* class, which was used without inclusion of discrete-geometric AuNP. Thereby the chemical compositions tabulated in Table 2 were used for the different parts of the cell. The gold was added to the cytosol. Gold mass percentages of 0.001%, 0.01%, 0.1%, 1.0% were simulated. Each simulation was repeated ten times with different seeds for the random number generator to allow for the calculation of the sample standard deviation, as reported in the following. Here, the position of each radioactive decay was chosen randomly within the cytosol.

### Properties of radioactive gold nanoparticles

The properties of radioactive AuNP, especially the influence of diameter and clustering behavior on the energy deposits characteristics were described in detail in our previous work^15^. Briefly, ^198^Au has an isotope mass of 198 u and a half life of 2.7  days. With a specific activity of 9.03$\times$10^15^ Bq/g, it performs a $\beta ^-$ decay with particle energies of 961 keV (99%) and 285 keV(1%) respectively, as well as $\gamma$ emissions with energies of 412 keV (96%), 676 keV (<1%) and 1088 keV (<1%) to the stable isotope ^198^Hg^61^. The range of the $\beta$ particles lies around (0.2-5) mm^62^. By adjusting the ratio of radioactive ^198^Au to non-radioactive ^197^Au within the AuNP, the individual activity of the AuNP, and therefore the delivered dose, can be adjusted to some extend independently from the number and AuNP radius itself. This can be achieved by parameters of the activation procedure^64^. Details on AuNP synthesis and the activation by neutrons can be found in^63,64^.

## Data Availability

The data that support the findings of this study are available from the corresponding author upon request.

## References

[1] Kuncic Z, Lacombe S (2017). Nanoparticle radio-enhancement: principles, progress and application to cancer treatment. Phys. Med. Biol..

[2] Letfullin RR, George TF (2017). Computational Nanomedicine and Nanotechnology: Lectures with Computer Practicums.

[3] McMahon SJ (2011). Nanodosimetric effects of gold nanoparticles in megavoltage radiation therapy. Radiother. Oncol..

[4] McNamara AL (2016). Dose enhancement effects to the nucleus and mitochondria from gold nanoparticles in the cytosol. Phys. Med. Biol..

[5] Sung W (2017). Dependence of gold nanoparticle radiosensitization on cell geometry. Nanoscale.

[6] Bahreyni Toossi MT (2012). A Monte Carlo study on tissue dose enhancement in brachytherapy: a comparison between gadolinium and gold nanoparticles. Australas. Phys. Eng. Sci. Med..

[7] Clement S, Deng W, Camilleri E, Wilson BC, Goldys EM (2016). X-ray induced singlet oxygen generation by nanoparticle-photosensitizer conjugates for photodynamic therapy: determination of singlet oxygen quantum yield. Sci. Rep..

[8] Schürmann R, Bald I (2017). Effect of adsorption kinetics on dissociation of DNA-nucleobases on gold nanoparticles under pulsed laser illumination. Phys. Chem. Chem. Phys..

[9] Letfullin RR, Iversen CB, George TF (2011). Modeling nanophotothermal therapy: kinetics of thermal ablation of healthy and cancerous cell organelles and gold nanoparticles. Nanomed. Nanotechnol. Biol. Med..

[10] Zharov VP, Letfullin RR, Galitovskaya EN (2005). Microbubbles-overlapping mode for laser killing of cancer cells with absorbing nanoparticle clusters. J. Phys. D Appl. Phys..

[11] Katti KV (2006). Hybrid gold nanoparticles in molecular imaging and radiotherapy. Czechoslov. J. Phys..

[12] Chanda N (2010). Radioactive gold nanoparticles in cancer therapy: therapeutic efficacy studies of GA-198AuNP nanoconstruct in prostate tumor- bearing mice. Nanomed. Nanotechnol. Biol. Med..

[13] Katti KV (2016). Renaissance of nuclear medicine through green nanotechnology: functionalized radioactive gold nanoparticles in cancer therapy- my journey from chemistry to saving human lives. J. Radioanal. Nucl. Chem..

[14] Laprise-Pelletier M, Simão T, Fortin M-A (2018). Gold nanoparticles in radiotherapy and recent progress in nanobrachytherapy. Adv. Healthc. Mater..

[15] Zutta Villate JM, Hahn MB (2019). Radioactive gold nanoparticles for cancer treatment. Eur. Phys. J. D.

[16] Gholami YH, Maschmeyer R, Kuncic Z (2019). Radio-enhancement effects by radiolabeled nanoparticles. Sci. Rep..

[17] McMahon SJ (2011). Biological consequences of nanoscale energy deposition near irradiated heavy atom nanoparticles. Sci. Rep..

[18] Schürmann R, Vogel S, Ebel K, Bald I (2018). The physico-chemical basis of DNA radiosensitization: implications for cancer radiation therapy. Chem. A Eur. J..

[19] Hahn MB (2017). DNA protection by ectoine from ionizing radiation: molecular mechanisms. Phys. Chem. Chem. Phys..

[20] Hahn MB (2017). Direct electron irradiation of DNA in a fully aqueous environment. Damage determination in combination with Monte Carlo simulations. Phys. Chem. Chem. Phys..

[21] Tran HN (2016). Geant4 Monte Carlo simulation of absorbed dose and radiolysis yields enhancement from a gold nanoparticle under MeV proton irradiation. Nucl. Instrum. Methods Phys. Res. Sect. B Beam Interact. Mater. Atoms.

[22] Hahn MB, Smales GJ, Seitz H, Solomun T, Sturm H (2020). Ectoine interaction with DNA: influence on ultraviolet radiation damage. Phys. Chem. Chem. Phys..

[23] Hainfeld JF, Slatkin DN, Smilowitz HM (2004). The use of gold nanoparticles to enhance radiotherapy in mice. Phys. Med. Biol..

[24] Shi J, Xiao Z, Kamaly N, Farokhzad OC (2011). Self-assembled targeted nanoparticles: evolution of technologies and bench to bedside translation. Acc. Chem. Res..

[25] Sykes EA, Chen J, Zheng G, Chan WC (2014). Investigating the impact of nanoparticle size on active and passive tumor targeting efficiency. ACS Nano.

[26] Chithrani BD, Ghazani AA, Chan WCW (2006). Determining the size and shape dependence of gold nanoparticle uptake into mammalian cells. Nano Lett..

[27] Özçelik S, Pratx G (2020). Nuclear-targeted gold nanoparticles enhance cancer cell radiosensitization. Nanotechnology.

[28] Schuemann J (2019). TOPAS-nBio: an extension to the TOPAS simulation toolkit for cellular and sub-cellular radiobiology. Radiat. Res..

[29] Agostinelli S (2003). Geant4: a simulation toolkit. Nucl. Instrum. Methods Phys. Res. Sect. A Accel. Spectrom. Detect. Assoc. Equip..

[30] Bernal MA (2015). Track structure modeling in liquid water: a review of the Geant4-DNA very low energy extension of the Geant4 Monte Carlo simulation toolkit. Phys. Med..

[31] Incerti S (2010). Comparison of GEANT4 very low energy cross section models with experimental data in water. Med. Phys..

[32] Perl J, Shin J, Schumann J, Faddegon B, Paganetti H (2012). TOPAS: an innovative proton Monte Carlo platform for research and clinical applications. Med. Phys..

[33] Allison J (2016). Recent developments in Geant4. Nucl. Instrum. Methods Phys. Res. Sect. A Accel. Spectrom. Detect. Assoc. Equip..

[34] Hahn MB (2020). TOPAS Cell Model with Nanoparticles.

[35] Hahn, M. B. https://github.com/BAMresearch/TOPAS-CellModels. Bundesanstalt für Materialforschung und -prüfung (2020).

[36] Matsuya Y (2018). Investigation of dose-rate effects and cell-cycle distribution under protracted exposure to ionizing radiation for various dose-rates. Sci. Rep..

[37] Kirkby C, Ghasroddashti E (2015). Targeting mitochondria in cancer cells using gold nanoparticle-enhanced radiotherapy: a Monte Carlo study. Med. Phys..

[38] Chithrani DB (2010). Gold nanoparticles as radiation sensitizers in cancer therapy. Radiat. Res..

[39] Mi Y, Shao Z, Vang J, Kaidar-Person O, Wang AZ (2016). Application of nanotechnology to cancer radiotherapy. Cancer Nanotechnol..

[40] Margis S (2020). Microdosimetric calculations of the direct DNA damage induced by low energy electrons using the Geant4-DNA Monte Carlo code. Phys. Med. Biol..

[41] Hahn MB, Meyer S, Kunte H-J, Solomun T, Sturm H (2017). Measurements and simulations of microscopic damage to DNA in water by 30 keV electrons: a general approach applicable to other radiation sources and biological targets. Phys. Rev. E.

[42] Lewinski N, Colvin V, Drezek R (2008). Cytotoxicity of nanoparticles. Small.

[43] Alkilany AM, Murphy CJ (2010). Toxicity and cellular uptake of gold nanoparticles: what we have learned so far?. J. Nanopart. Res..

[44] Metz O, Stoll W, Plenert W (1982). Meningosis prophylaxis with intrathecal 198Au-colloid and methotrexate in childhood acute lymphocytic leukemia. Cancer.

[45] Nikjoo DH, Emfietzoglou D, Charlton DE (2008). The Auger effect in physical and biological research. Int. J. Radiat. Biol..

[46] Lazarakis P (2018). Investigation of track structure and condensed history physics models for applications in radiation dosimetry on a micro and nano scale in Geant4. Biomed. Phys. Eng. Express.

[47] Emfietzoglou D, Papamichael G, Nikjoo H (2017). Monte Carlo electron track structure calculations in liquid water using a new model dielectric response function. Radiat. Res..

[48] Sakata D (2016). An implementation of discrete electron transport models for gold in the Geant4 simulation toolkit. J. Appl. Phys..

[49] Sakata D (2018). Geant4-DNA track-structure simulations for gold nanoparticles: the importance of electron discrete models in nanometer volumes. Med. Phys..

[50] Sakata D (2019). Electron track structure simulations in a gold nanoparticle using Geant4-DNA. Phys. Med..

[51] Basaglia T (2015). Investigation of Geant4 simulation of electron backscattering. IEEE Trans. Nucl. Sci..

[52] Byrne H, McNamara A, Kuncic Z (2018). Impact of nanoparticle clustering on dose radio-enhancement. Radiat. Prot. Dosim..

[53] Francis Z, Incerti S, Karamitros M, Tran HN, Villagrasa C (2011). Stopping power and ranges of electrons, protons and alpha particles in liquid water using the Geant4-DNA package. Nucl. Instrum. Methods Phys. Res. Sect. B Beam Interact. Mater. Atoms.

[54] Kyriakou I (2019). Influence of track structure and condensed history physics models of Geant4 to nanoscale electron transport in liquid water. Phys. Med..

[55] Dahm-Daphi C, Sass W, Alberti J (2000). Comparison of biological effects of DNA damage induced by ionizing radiation and hydrogen peroxide in CHO cells. Int. J. Radiat. Biol..

[56] Xu X (2011). The genomic sequence of the Chinese hamster ovary (CHO)-K1 cell line. Nat. Biotechnol..

[57] Ross D, Mel H (1972). Growth dynamics of mitochondria in synchronized Chinese hamster cells: ScienceDirect. Biophys. J..

[58] Peng J-Y (2011). Automatic morphological subtyping reveals new roles of caspases in mitochondrial dynamics. PLOS Comput. Biol..

[59] Salimi E, Braasch K, Butler M, Thomson DJ, Bridges GE (2016). Dielectric model for Chinese hamster ovary cells obtained by dielectrophoresis cytometry. Biomicrofluidics.

[60] White, D. R., Booz, J., Griffith, R. V., Spokas, J. J. & Wilson, I. J. Report 44. *J. Int. Comm. Radiat. Units Meas.* **os23**, NP–NP. 10.1093/jicru/os23.1.Report44 (1989).

[61] Delacroix D, Guerre PJ, Leblanc P, Hickman C (2002). Radionuclide and radiation protection data handbook 2002. Radiat. Protect. Dosim..

[62] Plante I, Cucinotta FA (2009). Cross sections for the interactions of 1 eV–100 MeV electrons in liquid water and application to Monte-Carlo simulation of HZE radiation tracks. New J. Phys..

[63] Kimling J (2006). Turkevich method for gold nanoparticle synthesis revisited. J. Phys. Chem. B.

[64] Zutta Villate, J. M., Rojas, J. V., Hahn, M. B. & Puerta, J. A. Synthesis and optimization of radioactive gold nanoparticles for cancer therapy. *J. Radioanal. Nucl. Chem.* **Manuscript in preparation** (2021).

